# Memory, Executive Skills, and Psychosocial Phenotype in Children with Pharmacoresponsive Epilepsy: Reactivity to Intervention

**DOI:** 10.3389/fneur.2017.00086

**Published:** 2017-04-21

**Authors:** Yael Schaffer, Bruria Ben Zeev, Roni Cohen, Avinoam Shufer, Ronny Geva

**Affiliations:** ^1^Department of Psychology, Bar-Ilan University, Ramat Gan, Israel; ^2^The Gonda Multidisciplinary Brain Research Center, Bar-Ilan University, Ramat Gan, Israel; ^3^The Neurology Department at Sheba Medical Center, Ramat Gan, Israel; ^4^Department of Pediatric Neurology and Epilepsy Center, Schneider Children’s Medical Center of Israel, Petach Tikva, Israel; ^5^Sackler Faculty of Medicine, Tel Aviv University, Tel Aviv, Israel

**Keywords:** pediatric pharmacoresponsive epilepsies, verbal memory, short-term memory, psychosocial, rehabilitation, memory intervention

## Abstract

**Background:**

Recent studies on pharmacoresponsive epilepsies demonstrate specific memory, executive functions (EF), and psychosocial deficits in this group. These deficits are often undertreated, and little is known about the neuropsychological factors that may support moderation of the deficits through intervention. The aim of this study was to explore the effects of a structured cognitive behavioral group intervention on both memory and emotional domains and to evaluate the factors influencing its efficacy.

**Methods:**

The feasibility study implemented a newly designed intervention for children with pharmacoresponsive epilepsies (*N* = 33, aged 9–14 years, 51% girls), hypothesizing that memory and psychosocial symptoms in children with pharmacoresponsive epilepsies are sensitive to intervention using structured memory and psychosocial modules in a weekly group session setting. Comparable memory and psychosocial assessments were used to evaluate performance at baseline and post-intervention. Results were compared to age- and education-matched healthy controls (*N* = 27, aged 9–14 years).

**Results:**

Pre–post-intervention comparisons show improvements in STM (*p* < 0.01, η^2^ = 0.358), optimism (*p* < 0.05, η^2^ = 0.245), and self-efficacy (*p* < 0.05, η^2^ = 0.164). Unique negative relations between memory deficits and psychosocial phenotype were seen in epilepsy patients and not in controls in response to the intervention. EF moderated this intervention effect (*p* < 0.05, η^2^ = 0.252), whereas psychosocial status and pharmacological profile did not.

**Conclusion:**

Cognitive behavioral therapy focusing on memory and psychosocial perceptions for children with pharmacoresponsive epilepsies seems promising, with greater improvement in memory and psychosocial functioning in children with more affected EF.

## Introduction

Epilepsy, a group of disorders characterized by disturbances in electrical signaling in the brain, is quite common in the general population (1, 2). According to the International League Against Epilepsy (ILAE) Commission on classification and terminology (3), one-third of all patients with epilepsy suffer from genetically determined epilepsies, such as childhood absence epilepsy, or epilepsies from unknown cause, such as self-limited rolandic epilepsy (3). These epilepsies, formerly known as “idiopathic” epilepsy, often affect children and usually respond to antiepileptic drugs (AEDs). In our study, we focused on pharmacoresponsive epilepsies of childhood that are genetically determined or from an unknown cause (3). Pharmacoresponsive epilepsies of childhood often spontaneously remit during predictable age ranges and are often thought to be unaccompanied by other consequences or disabilities (4).

Recently, studies noted a variety of subtle cognitive and behavioral disorders associated with these epilepsies (3, 5). More specifically, discrete memory dysfunction and executive functions (EF) deficits were found in children with pharmacoresponsive epilepsies (5–9).

Memory and EF are crucial for learning in children (10). The specific neuropsychological profile includes short-term memory deficits (STM; auditory, verbal, and visual), working memory (WM), and auditory verbal long-term memory (LTM) deficits with preserved long-term visual memory skills (5, 7, 9, 11, 12). The cluster of deficits points to a modality issue, underscoring a particular sensitivity in coding and memorizing auditory verbal material as compared with non-verbal or visual stimuli (5, 8, 11, 13).

Although the causes of memory impairments in patients with epilepsy have not been completely elucidated, several factors are considered to be involved. Basic neurophysiological work suggests that seizures may modify, slow down, or accelerate processes that take place during development; processes that are essential for the intact formation and function of neural circuitry (14, 15). The underlying etiology of epilepsy may cause memory impairments (16). As such, these memory deficits also represent the epiphenomena of a dysfunctional cortex (17).

The tight brain–behavior relationship is well reflected in cognitive functionals as well as on the effects of AEDs on the central nervous system. There are two main approaches for treating epilepsy pharmacologically. The first, by using monotherapy; the second, by using polytherapy.

Some AEDs have a side effect on cognitive function, with polytherapy resulting in more cognitive side effects than in monotherapy (18, 19). In addition, EF that play significant roles in memory functions (20, 21) and in memory rehabilitation (12) frequently demonstrate deficits in pharmacoresponsive epilepsies (especially absence and self-limited epilepsy of childhood) (12, 21, 22).

Executive function deficits include deficits in goal-directed activity, planning, and self-regulation of behavior (7, 23–26); functions that are potentially linked to the child’s ability to retrieve information from memory in manners relevant for their cognitive performance. EF role may be particularly relevant in children with pharmacoresponsive epilepsies, as EF has a protracted developmental course paralleling functional maturity of the frontal networks in late childhood (27).

In this study, we explored the notion that there is a possible mediating role of EF and metacognitive abilities in supporting memory in middle childhood (28, 29). The mediating role of EF may then be evident in the implementation of cognitive interventions that teach mental strategies and information updating techniques.

Until recently, research on the effectiveness of memory rehabilitation techniques on pediatric patients with known neurological involvement was scarce (30–32). In the past 8 years, however, five reviews of cognitive interventions in children were conducted (12, 31–34), as well as one meta-analysis (35), and one review on the applicability of cognitive rehabilitation for children with acquired brain injury (ABI) (36).

These studies included relatively few studies specifically addressing memory deficits as the target for cognitive rehabilitation. It seems important to address this literature gap in view of the role of memory in school functioning (7, 37); by activating autobiographical memories (i.e., recalling personally experienced events) and enabling drawing inferences concerning the mental states of others (i.e., mentalization or theory of mind) (38–41). Also, these studies focused on a pediatric population with ABI. As far as we know, no memory rehabilitation for children with epilepsy was published yet (12). Therefore, we thought that focusing on memory rehabilitation on this understudied population is important.

Although cognitive effects dominate the literature on childhood pharmacoresponsive epilepsies, there are also reports of selected social and behavioral outcomes in this population. Children with pharmacoresponsive epilepsies were found to have higher rates of depression, anxiety, lower self-esteem, psychosis, and behavioral problems than in the general population (42–44). These data highlight the lower socioemotional functioning in children with pharmacoresponsive epilepsies in addition to their reported memory difficulties, possibly suggesting interrelations between these domains.

Recent research notes relationships between psychosocial symptoms and auditory verbal memory (AVM) in children with pharmacoresponsive epilepsies (5), and between AVM and social symptoms in this group (45). This relationship between psychosocial symptoms and AVM may suggest psychosocial functioning as a potential path that may moderate memory intervention in pharmacoresponsive epilepsies patients.

To best reach the target population, we developed a CBT protocol in a group format that provides a supportive social setting. Group interventions are thought of as an effective intervention, designed to provide exposure to coping/problem-solving skills, facilitate an environment with peer support, and help mitigate maladaptive strategies (46). CBT interventions in populations with epilepsy have thus far focused on coping with stigma associated with the disorder (47); and improving the patient’s sense of well-being (48), yet studies with the aim of addressing memory deficits in addition to psychiatric deficits through intervention have not yet been conducted.

In our current research, we developed a brief 10 group session clinical training intervention (about 15 total child-training hours) that is designed to teach strategies that promote planning, metacognition, and organization along with elaborative encoding strategies to improve AVM in daily functioning, with an emphasis on self-monitoring and self-efficacy.

**Figure 1:**
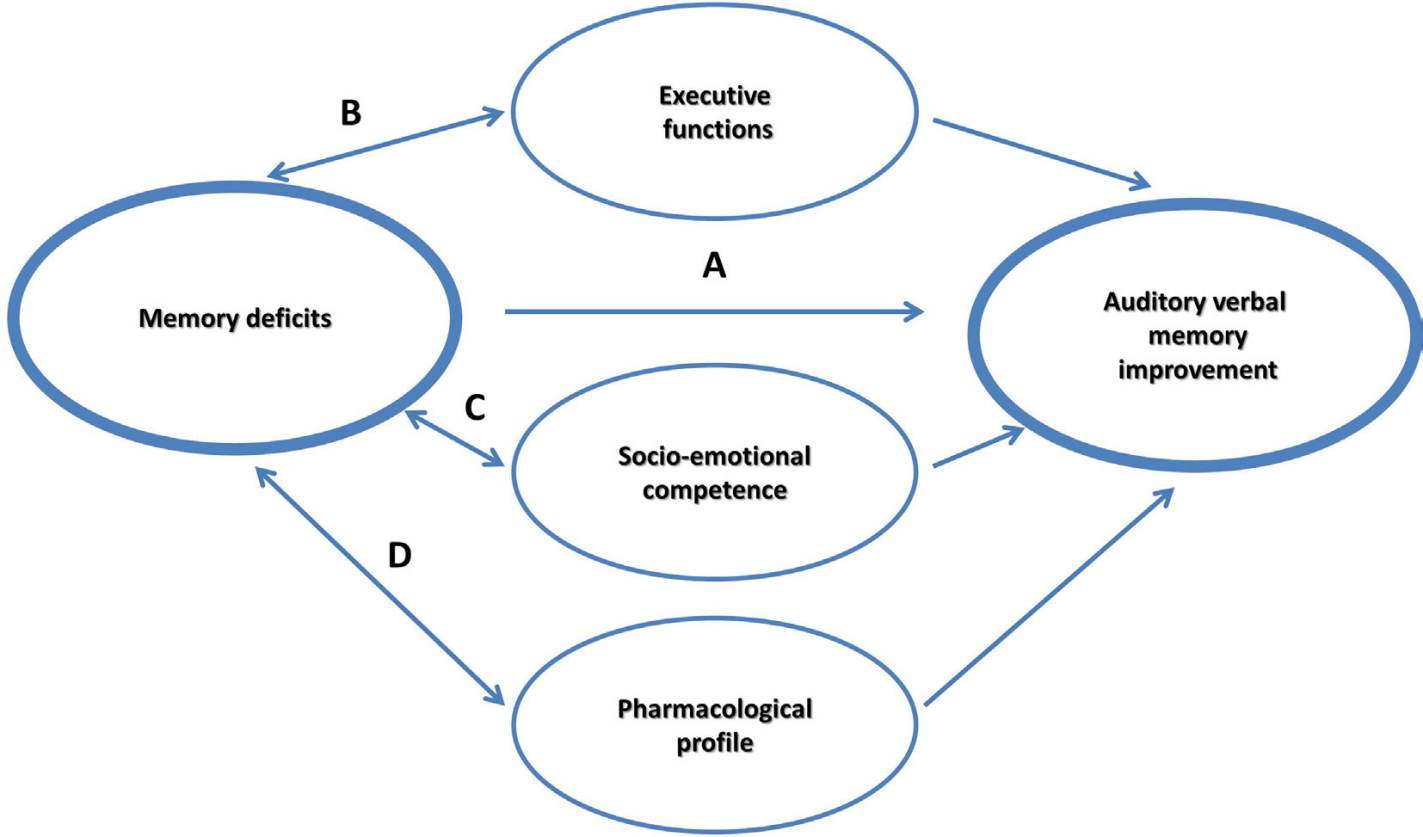
**Model for memory improvement in childhood pharmacoresponsive epilepsies**.

It is currently debated if memory itself can be improved as a result of practicing strategies; or rather CBT strategies influence mediating factors, such as supporting self-esteem, and training executive control. The main objective of the study was to examine whether an intervention will improve AVM, EF, self-coping, and self-efficacy in children with pharmacoresponsive epilepsy. In the current framework, we explored four hypothesized non-mutually exclusive effects that may account for the possible improvement in the most susceptible memory component in this group, the AVM (see Figure 1):

(a) A main intervention effect on AVM.
(b) A moderation effect through supporting EF.
(c) A moderation effect through supporting socioemotional competence, by encouraging an optimistic outlook and a strengthened sense of self-efficacy.
(d) A moderation effect by the pharmacological intervention that is prescribed to prevent seizures.

## Materials and Methods

### Participants

Children with pharmacoresponsive epilepsies were enrolled through the neurology departments at Sheba Hospital, Tel Hashomer Hospital, and Schneider Children’s Hospital at the Rabin Medical Centre, Israel. Participants consisted of 60 children: 33 were diagnosed with pharmacoresponsive epilepsies (mean age M = 10.88, SD = 1.5; 51% females); 27 healthy control participants (mean age M = 10.18, SD = 1.4; 52% females).

Inclusion was based on a clinical neurological review of patient’s medical history, electroencephalography, and imaging data by the treating neurologists according to the ILAE criteria (3, 49). Inclusion criteria for this group consisted of at least one unprovoked non-febrile seizure or status epilepticus in the past but no current seizures (i.e., balanced condition) for at least 1 year; pharmacoresponsive epilepsies that include genetically determined etiology and unknown etiology; self-limited rolandic epilepsy (*n* = 18), and genetic generalized epilepsies (*n* = 15) including absence epilepsy (*n* = 4). All children were fluent in their native language, Hebrew; with an estimated intelligence within normal limits (ESIQ > 79) based on the WISC-IV Block Design subtest (50); and attended mainstream schools.

Exclusion criteria included temporal epilepsy, structural epilepsy, metabolic epilepsy, comorbid neurological disorders other than ADHD (51), history of head injury, comorbid chronic illness (e.g., diabetes and asthma), major depression (51), psychosis, and prescribed use of topiramate and phenobarbital AEDs, which are known to compromise memory.

Overall, all the children that fulfilled the inclusion and exclusion criteria and committed to participate in a weekly clinical intervention at Bar-Ilan University were referred to the study by their neurologists. The referring neurologists were not aware of the specific objectives of this study or its hypotheses.

Six of the 39 referred children were excluded, four because of low intelligence and two because of the presence of more severe epilepsy (e.g., temporal lobe epilepsy or Lennox–Gastaut syndrome). Also, six children were removed from the study as they did not show up for the post-intervention assessment. Participants were divided randomly into groups. Overall, there were three groups for children of age 9–11 and three groups for children of age 12–14.

Comparisons between these participants and healthy participants showed that their baseline memory and psychosocial performance were comparable [memory function (*T*_31_ = −0.398, *p* = N.S.); self-efficacy (*T*_31_ = −1.766, *p* = N.S.)]. The demographics, seizure characteristics, and medication profiles of the participants are summarized in Table 1.

**Table 1:** **Participant demographics, seizure characteristics, and medication profile**.

	Epilepsy	Controls	Test of sig.	*p*-Value
*N*	33	27		
Age	10.88 ± 1.52	10.18 ± 1.4	*T*_58_ = 1.83	0.072 NS
Education level (years)	5.69 ± 1.158	5.55 ± 1.154	*T*_58_ = 0.471	0.639 NS
Age at diagnosis	6.86 ± 2.98	N/A		
Epilepsy duration	4.53 ± 2.46			
Education level^a^	9/10/8/5/1	11/8/6/1/2	$\chi_{4}^{2}=4.611$	0.330 NS
Socioeconomic status^b^	6.727 ± 1.12	6.296 ± 0.72	*T*_58_ = 1.718	0.091 NS
Sex (F/M)	17/16	14/13	$\chi_{1}^{2}=0.01$	0.974 NS
ESIQ	−0.85 ± 0.84	0.16 ± 0.93	*T*_58_ = −3.87	<0.01
Medication profile (%)^c^	75/25	0/0		
Psychotherapy	10	0	$\chi_{1}^{2}=16.63$	<0.01
Comorbid ADHD/LD	6/4	0/0	$\chi_{1}^{2}=9.9$	<0.02
Epilepsy type (%)^d^	18/15/4	0/0/0		

*Estimated intelligence (ESIQ), block design subtest from the WISC-IV; ADHD, attention deficit and hyperactivity disorder; LD, learning deficits*.

*^a^Fourth grade/fifth grade/sixth grade/seventh grade/eighth grade*.

*^b^According to the Central Bureau of Statistics in Israel (grades from 1 to 10)*.

*^c^Monotherapy/polytherapy*.

*^d^Self-limited epilepsy, genetic generalized epilepsies, and absence epilepsy*.

The control participants (27 children; 14 females and 13 males), matched for age and sex to the experimental group, were randomly recruited from mainstream public schools in the same (central) district of Israel *via* word-of-mouth (snowball recruitment) and were exposed to the same educational curriculum as the experimental group. Past medical history, as reported by their parents, was non-remarkable. The same inclusion criteria that applied to the epilepsy group were also used for this group with regard to age, intelligence, reported head injuries, schooling, psychiatric involvement, and medication.

The study was approved by the Institutional Review Boards of the participating medical centers: Sheba Hospital, Tel Hashomer (7738-10-SMC); Schneider Children’s Medical Centre, Petach Tikva (TLV-0282-11), and Bar-Ilan University, Israel, in accordance with the guidelines of the Helsinki Declaration. Written informed consent was obtained from all parents, and oral consent was obtained from the participants before participating in the intervention.

### Intervention Design

The complete intervention program for the pharmacoresponsive epilepsies group consisted of two 5-week modules: a Memory Skills Training module and a Psychosocial Training Module (52). Efficacy was tested using two sessions with comparable, yet not identical items, to limit exposure effects (Table 2). The pre-training assessment provided baseline measures, and the second assessment occurred after training was completed. The intervention sessions were administered to groups of 4–6 participants that were carefully matched for age and genders. “Homework” task completion was monitored between sessions, employing parental mediation *via* emails, to maximize efficacy and ensure comparability. Further details concerning the intervention were recently published (52).

**Table 2:** **Neuropsychological and psychosocial assessment tools**.

Domain	Auditory	B	PI	Visual	B	PI
Intelligence	WISC-IV: vocabulary	*		WISC-IV: block design	*	
STM	TOMAL: paired recall	*	V2	TOMAL: facial memory	*	*
	Digit forward	*	V2	Abstract visual memory	*	*
	Object recall		*	Visual sequential memory	*	*
	Word selective memory		*	Memory for location	*	*
	Immediate memory for stories	*				
	CMS immediate					
	RAVLT: immediate	*				
Long-term memory (LTM)	TOMAL: delayed memory for stories	*		RCFT: immediate and delayed visual memory	*	*
	Word selective memory		*			
	CMS delayed memory	*	*			
	RALVT: delayed					
	Recognition					
Working memory	TOMAL: digits backwards	*	V2	WISC-IV: cancelation	*	*
				Number canceling		
Executive functions	NEPSY: inhibition (53)	*	*	NEPSY: animal sorting	*	*
Total psychosocial functioning	CBCL (parents)	*	*			
	YSR (child)	*	*			
Self-efficacy	GSE	*	*			
Optimism	CSC	*	V2			
	YLOT	*	*			

*B, baseline assessment; PI, post-intervention assessment; VS, version 2; WISC-IV, Wechsler intelligence scale for children IV addition (50); TOMAL, test of memory and learning (54); CMS, child memory scale (55); RALVT, Rey auditory verbal learning test (56); RCFT, Rey complex figure test (57); NEPSY, a developmental neuropsychological assessment (53); CBCL, Child Behavior checklist, parent’s form (58); YSR, youth self-report subtest (58); GSE, General Perceived Self-Efficiency scale (59); CSC, Children’s Self-Control scale (60); YLOT, Youth Life Orientation Test (61)*.

*Represents that the test was used and not a significance level.

#### Procedure for Healthy Controls

Control participants were tested individually at the lab in one 120 min session, with breaks as required. Developmental histories were gathered *via* parental interviews during separate sessions.

#### Memory Training

The Memory Training Intervention module focused on learning strategies and techniques to improve organizational and memory skills, such as chunking and mnemonic methods (62), self-awareness of individual memory slips, categorization, story making, visual imagery, and association (62, 63). The module is based on recent work in memory rehabilitation of children (64–66) and adults (67). Strategies, such as smartphone reminders and learning the importance of routines and habits, are practiced and encouraged as well (68, 69). For a description of the intervention, detailed in Schaeffer and Geva (52).

#### Psychosocial Support Setting

This module concentrates on several core issues in children with pharmacoresponsive epilepsies, including personal feelings associated with epilepsy, self-efficacy beliefs, coping strategies, and optimism regarding future challenges (46). The module is based on recent psychosocial interventions for children with learning disorders (70), epilepsy (46), depression (71, 72), and adults with normal aging (73). The Psychosocial Training Module introduces coping strategies in a similar way as other coping intervention models (74). Each coping dimension (belief, affect, social, imagination, cognition, and physiology) was introduced and practiced along with additional strategies that were incorporated from classic CBT protocols (71, 75) designed to expand the participants’ sense of self-efficacy.

### Pre- and Post-Neuropsychological Evaluations

Comparable extensive age-appropriate assessment batteries were used. The batteries included standardized tests of STM, LTM, and WM, as well as tests for emotional and social problems, to explore baseline and intervention outcome on tasks and stimuli. Careful attention was given to test the trained capacities yet avoid testing items that were directly tested initially or practiced in the intervention sessions. Two comparable versions of verbal STM and LTM neuropsychological tests and Children’s Self-Control (CSC) scale (76) were used to limit practice effects and task familiarity. Clinical neuropsychological instruments are summarized in Table 2.

Four research assistants participated in the study to ensure a blind research paradigm by independent assignment of random identification codes, conducting evaluations of participants with the standard methods and checking scoring test results to limit errors. About a month passed between baseline assessment and initiation of the intervention protocol. Posttreatment assessments took place within 1–2 weeks from the end of the last group session.

### Statistical Approach

#### Memory Outcome Analysis

To examine the hypothesis that intervention will affect AVM more than visual memory, average verbal and visual memory scores were computed and included in an ANOVA with repeated measures, analyzing memory score as a function of modality (auditory versus visual memory) and intervention (baseline versus post-intervention assessment). To explore effects on the three attention networks, a 2 × 3 repeated measures ANOVA was computed comparing individual memory system scores (STM, WM, and LTM) as a function of intervention.

To investigate the frequency of treated patients with normalized performance before and after the intervention, two chi-square analyses were conducted, testing the relation between typical versus deficient auditory verbal STM (using a cut-off of *Z* score <−1) and group (epilepsy versus healthy controls) at baseline and post-intervention. Odds ratios for risk were then computed.

#### Psychosocial Analysis

To examine the notion that intervention affects self-efficacy and optimism, two ANOVAs with repeated measures were performed, with intervention as a within-subjects factor and psychosocial subtest scores as dependent measures. Two chi-square tests were then conducted to measure propensity for deficits in these dimensions pre- and post-intervention, with *Z* score <−1 as the criterion for the existence of a deficit. Odds ratio for risk was calculated. In the next step, we examined the relationships between memory domains and psychosocial status at baseline (5) and at posttreatment. Differences in correlation strengths between the pharmacoresponsive epilepsies and control groups were explored using the Fisher *Z* coefficient (77, 78).

#### Moderation Effects

To explore mediating roles of baseline EF (high/low determined by median split), optimism (high/low determined by median split), or self-efficacy score (high/low determined median split), in memory outcome, three ANOVAs with repeated measures were run, with EF, optimism and self-efficacy as the within-subject variables and modality and intervention as dependent measures. Finally, to evaluate the role of the pharmacological profile (monotherapy versus polytherapy), all moderation analyses were calculated with the pharmacological profile as a covariate.

## Results

### Memory Outcome

To test whether intervention affects AVM more than visual memory, an ANOVA with repeated measures was conducted comparing memory scores as a function of modality and intervention. Findings showed a significant modality effect [*F*_(1, 26)_ = 29.192, *p* < 0.01, η^2^ = 0.529], such that AVM was lower (M = −1.3, SD = 0.53) relative to visual memory (M = −0.6, SD = 0.83); a significant intervention effect [*F*_(1, 26)_ = 9.105, *p* < 0.01, η^2^ = 0.259], such that both modalities improved after the intervention. No modality × intervention interaction was seen.

**Figure 2:**
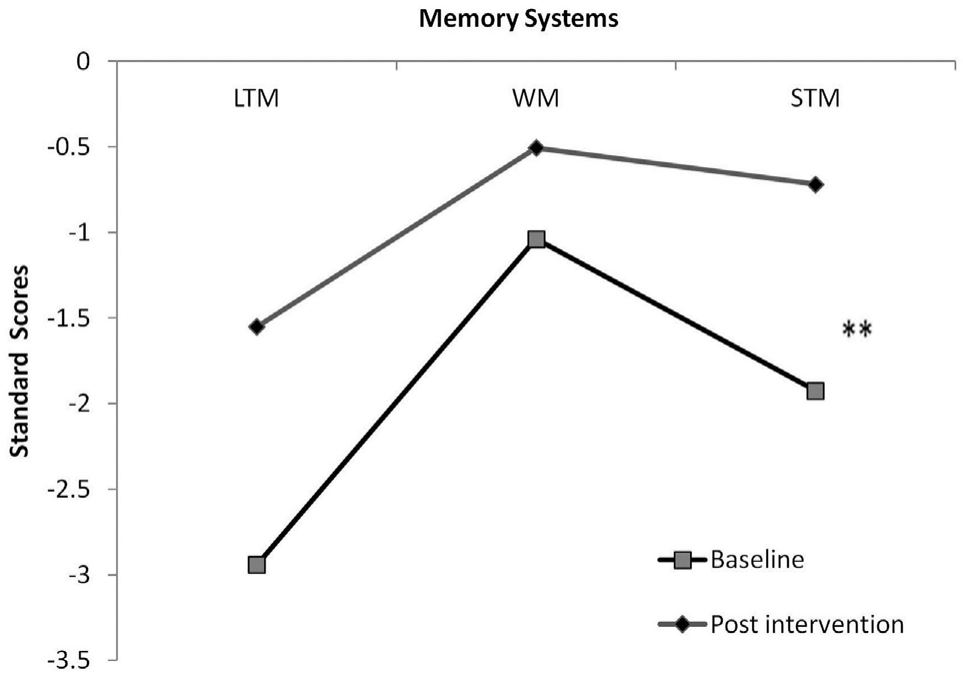
**STM, short-term memory; WM, working memory; LTM, long-term memory**. **p < 0.01.

To deepen the exploration of proposed path *a*, we included STM, LTM, and WM scores in a repeated measures analysis, comparing memory systems pre- and post-intervention. Results showed a moderate effect for memory system [*F*_(1, 26)_ = 8.713, *p* < 0.002, η^2^ = 0.492] and a near significant interaction between memory system and intervention effect [*F*_(1, 26)_ = 3.178, *p* = 0.06, η^2^ = 0.261]. Hypothesis-driven *post hoc* examination highlighted a post-intervention improvement only in STM (*p* < 0.01) (Figure 2). To explore the specificity of the effect, intelligence was included as a covariate in the above analysis. Results revealed preserved effects.

#### Memory Systems Interaction Effect

Chi-square analysis relating the frequency of normalized auditory AVM performance and group (epilepsy versus Controls) showed a difference in memory susceptibility at baseline [χ^2^_(1, 58)_ = 13.806, *p* < 0.01], with 14 children (42.4%) in the epilepsy group and 24 children (88%) in the control group displaying auditory verbal STM within normal range (odds ratio10.857; 95% confidence interval: 2.719–43.355; *p* < 0.001). Results from this analysis post-intervention remained significant [χ^2^_(1, 52)_ = 8.584, *p* < 0.01], now with 16 children (53.3%) in the epilepsy group showing auditory verbal STM within normal range, leading to reduced odds ratios for risk of auditory verbal STM deficits post-intervention (odds ratio 7; 95% confidence interval: 1.729–28.336; *p* < 0.001).

A within-group chi-square test of independence revealed a significant propensity for performance within normal range post-intervention [χ^2^_(1, 58)_ = 4.159, *p* < 0.05], with 21.25% of the children in the epilepsy group shifting from deficient levels to normal range in AVM functioning, which was their most affected domain.

### Psychosocial Outcome

Three repeated measures analyses comparing optimism, self-efficacy, and total psychosocial outcome scores pre- and post-intervention were conducted. Results indicate modest intervention effects for both optimism [YLOT (79)] [*F*_(1, 26)_ = 7.454, *p* < 0.05, η^2^ = 0.245] and for self-efficacy [GSE (59)] [*F*_(1, 26)_ = 4.503, *p* < 0.05, η^2^ = 0.164], such that both were higher post-intervention. No differences were seen between total psychosocial outcome scores as a function of intervention.

#### Self-Efficacy, Optimism, and Psychosocial Outcome

Chi-square analyses comparing propensity for deficits in self-efficacy (high/low), optimism (high/low), and psychosocial outcome (high/low) were run at baseline and post-intervention. No significant differences were seen between pre- and posttesting for the children in the epilepsy group, though the number of children who scored within normal range did increase after intervention [e.g., 17 children (51%) reached normal range for self-efficacy compared to 13 (39%) at baseline assessment, NS].

Before testing each one of the moderation paths, we first examined whether participants with epilepsy had lower EF, self-efficacy, and optimism at baseline in comparison with healthy controls. Results showed group EF differences [*F*_(1, 58)_ = −24.195, *p* < 0.000], demonstrating lower EF in children with epilepsy (M = −1.237, SD = 1.01) as compared to controls (M = −0.0039, SD = 0.979). No differences were seen for self-efficacy or optimism.

#### EF Moderation Effect

To further explore the EF moderation hypothesis, an ANOVA with repeated measures was conducted with modality scores as a function of high/low EF scores and intervention. Findings showed a modality effect [*F*_(1, 26)_ = 26.061, *p* < 0.0001, η^2^ = 0.531] and a intervention effect [*F*_(1, 26)_ = 5.803, *p* < 0.05, η^2^ = 0.201]. Additionally a modality × intervention × EF effect was found [*F*_(1, 26)_ = 7.757, *p* < 0.05, η^2^ = 0.252], such that children with lower EF gained more from the intervention than those with higher EF particularly in verbal tasks. Finally, to explore the specificity of the EF moderation effect, intelligence was included as a covariate in the above analysis. Results revealed preserved effects.

#### Emotional Moderation Effect

To examine emotional moderation of memory outcome as a function of optimism or self-efficacy scores, comparable analyses as performed with psychosocial tests were used. Results for both showed modest to moderate effects on the affected auditory memory, as compared with the relatively preserved visual memory [optimism: *F*_(1, 26)_ = 7.064, *p* < 0.05. η^2^ = 0.235; self-efficacy: *F*_(1, 26)_ = 31.312, *p* < 0.01, η^2^ = 0.556] and intervention effects [optimism: *F*_(1, 26)_ = 27.337, *p* < 0.01, η^2^ = 0.543; self-efficacy: *F*_(1, 26)_ = 8.752, *p* < 0.01, η^2^ = 0.259, respectively]. No interactions were noted between socioemotional dimension and memory intervention outcomes.

Finally, to explore the specificity of the emotional moderation effect, intelligence was included as a covariate in the above analysis. Results revealed preserved effects.

#### Pharmacological Moderation Effect

A comparable repeated measures analysis, now with pharmacological profile (monotherapy/polytherapy) as a between-subjects variable, reaffirmed the moderate intervention effect [*F*_(1, 26)_ = 13.333, *p* < 0.001, η^2^ = 0.367], with no interaction between auditory verbal STM and pharmacological profile [*F*_(1, 26)_ = 0.191, *p* = 0.666, η^2^ = 0.008], indicating that pharmacological intervention does not account for further explained variance in this context.

#### Relationships between AVM and Psychosocial Outcome Posttreatment

A correlation matrix was constructed to examine relationships between AVM deficits and socioemotional status before and after treatment. Apparently the significant correlation between auditory verbal STM and total psychosocial functioning, which was found before treatment for the epilepsy group and not for the controls (5), was affected by the intervention and was now no longer significant (Table 3, *r* = −0.553, *p* < 0.21 for the epilepsy group; *r* = −0.132, *p* < 0.55 for the control group).

**Table 3:** **Correlations between psychosocial outcome problems and memory functions posttreatment**.

Psychosocial domain	Psychosocial symptoms	Dependent measures Auditory verbal STM	Pearson *r* in epilepsy group (*p*<) BT	Pearson *r* in epilepsy group (*p*<) PT	Pearson *r* in healthy control group	Fisher *Z*—BT	Fisher *Z*—PT	p *1-tailed **2-tailed
Int.	Anxiety	MF	**−**0.57 (0.006)	n.s.	−0.137 (0.54)	−1.64		BT: 0.05*
								0.1** NS
	Depression	DF	**−**0.44 (0.027)	n.s.	−0.226 (0.312)	−0.8		BT: 0.4 NS
		PR	n.s.	0.553 (0.021)			1.41	PT: 0.15 NS
		MFS	**−**0.5 (0.008)	n.s.	−0.03 (0.8)	−1.7		BT: 0.04*
								0.08 NS**

Ext.	Delinquent behavior	MFS	**−**0.387 (0.03)	n.s.	−0.007 (0.71)	−1.39		BT: o.16 NS
	Aggressive behavior	MFS	**−**0.406 (0.04)	n.s.	0.04 (0.85)	−1.52		BT: 0.12 NS

SP	Social problems	DF	**−**0.425 (0.021)	**−**0.579 (0.012)	0.135 (0.5)	−1.98	−2.32	BT: 0.0239*
								0.0477**
								PT: 0.0102*
								0.0203**
		MFS	**−**0.48 (0.01)	n.s.	−0.09 (0.66)	−1.43		BT: 0.15 NS

CD	Thought problems	MFS	**−**0.533 (0.005)	n.s.	−0.18 (0.4)	−1.33		BT: 0.18 NS
		DF	**−**0.47 (0.018)	n.s.	−0.345 (0.115)	−0.48		BT: 0.6 NS
	Attention	DF	**−**0.47 (0.018)	**−**0.653 (0.003)	0.258 (0.2)	−2.38	−3.06	BT: 0.0087*
								0.017**
								PT: 0.0011*
								0.0022**
		MFS	**−**0.556 (0.002)	n.s.	−0.12 (0.58)	−1.63		BT: 0.05*
								0.1 NS**

**Psychosocial domain**	**Psychosocial symptoms**	**Dependent measures Auditory verbal long-term memory**	**Pearson *r* in epilepsy group (*p*<) BT**	**Pearson *r* in epilepsy group (*p*<) PT**	**Pearson *r* in healthy control group BT**	**Fisher *Z*—BT**	**Fisher *Z*—PT**	p *1-tailed **2-tailed

Int.	Anxiety	MFSD	**−**0.52 (0.006)	n.s.	−0.407 (0.06)	−0.47		BT: 0.6 NS
		RALVT8	**−**0.429 (0.046)	n.s.	−0.017 (0.945)	−1.32		BT: 0.1868 NS
	Depression	MFSD	**−**0.408 (0.038)	n.s.	−0.27 (0.213)	−0.5		BT: 0.61 NS
	General	MFSD	**−**0.49 (0.009)	n.s.	**−**0.58 (0.004)	0.41		BT: 0.68 NS
		RALVT8	**−**0.43 (0.041)	n.s.	−0.17 (0.47)	−1.23		BT: 0.218 NS

Ext.	Delinquent behavior	MFSD	**−**0.58 (0.003)	n.s.	−0.118 (0.58)	−1.87		BT: 0.03*
								0.06** NS
		RALVT9	**−**0.564 (0.006)	n.s.	−0.003 (0.9)	−1.9		BT: 0.0287*
								0.057**
		RALVT8	**−**0.441 (0.04)	n.s.	−0.062 (0.79)	−1.29		BT: 0.1971 NS

SP	Social problems	MFSD	**−**0.247 (0.03)	n.s.	−0.358 (0.1)	−0.34		BT: 0.773 NS
		RALVT8	**−**0.447 (0.037)	n.s.	0.043 (0.856)	−1.57		BT: 0.058*
								0.116** NS

CD	Thought problems	MFSD	**−**0.516 (0.007)	n.s.	−0.36 (0.1)	−0.62		BT: 0.5353 NS
	Attention	MFSD	**−**0.468 (0.016)	n.s.	−0.162 (0.473)	−1.11		BT: 0.267 NS

*Int., introvert; Ext., extrovert; SP, social problems; CD, cognitive distractions; BT, before treatment; PT, posttreatment; STM, short-term memory*.

Similarly, correlations between auditory verbal LTM and total psychosocial functioning, which were significant before treatment only in the epilepsy group and not in the control group were no longer significant posttreatment (*r* = −0.447, *p* < 0.05 for the epilepsy group and *r* = −0.0.043, *p* < 0.856 for the control group). Further, relations previously seen in children with epilepsy between depression, anxiety, social problems, and immediate and delayed AVM were no longer significant after the intervention, except for a persistent relationship between immediate AVM and social problems (Table 3).

## Discussion

The current feasibility study aimed to examine the relevance of CBT rehabilitation program in a small heterogeneous group of children with pharmacoresponsive epilepsies. The study employed a small sample size and thus the results should be addressed with caution. Pending replication, the results support the notion that memory deficits and psychosocial symptoms in this population are sensitive to intervention. The focus of the current feasibility study was to suggest a new group CBT treatment for children with epilepsy and to study potential moderators that may play roles in neuropsychological performance and psychosocial symptoms in this understudied population.

Results indicate improvements in both auditory and visual memory domains, with greater gains in the auditory verbal domain. This AVM improvement was mostly evident in short-term memory tasks than in LTM functions. Also, improvements in psychosocial symptoms and total social performance were also noted. Indeed, recent studies have suggested that psychosocial status should be assessed and treated in this population in addition to AVM (5).

In this study, using a new CBT intervention that included 10 structured group sessions seemed to support improvement in optimism and self-efficacy; even though these findings were not found to play a role memory improvement itself. The intervention seemed to show the sensitivity of both memory and psychosocial dimensions to intervention, possibly suggesting the usefulness of intervention to grant a higher sense of well-being to children with pharmacoresponsive epilepsies. The psychosocial and memory improvement may result from the direct intervention as well as from the facilitation of carryover between sessions that included electronic communication (*via* emails and SMS) between participants, their parent, and the group leader (80). The results add important support to the view that addressing psychosocial factors in children with epilepsy may be beneficial in supporting their sense of control and well-being.

As memory issues along with psychosocial problems are core issues for several diagnostic children groups, it seems important to understand better the processes involved in these beneficial outcomes. We analyzed three non-mutually exclusive moderation effects on memory improvements (EF, socioemotional competence, and pharmacological profile).

Analysis showed that of the three effects, it was the EF moderation effect that was significant. More specifically, results showed that using a CBT intervention, which incorporates specific strategies to improve memory, using metacognitive strategies and strategies that are rooted in EF, does not necessarily improve EF. Apparently, EF may have supported memory intervention particularly in those with weaker EF skills, thereby supporting the EF moderation path in memory improvements in pharmacoresponsive epilepsies (Figure 1).

This finding may point to the notion that EF potentially plays a moderating role in CBT in pediatric populations. Contemporary research in neuropsychology, cognitive neuroscience, and clinical psychology has proposed that preserved EF facilitates the successful use of CBT in adults (81, 82).

Until recently, it was suggested that CBT might be less beneficial at young ages and in pediatric neurological populations, such as those with ADHD, given their immature EF abilities (83, 84). Current data demonstrate that in older children (9–14 years of age) who suffer from EF deficits (7, 23–26), and in children suffering from anxiety and depression (72, 75) CBT can be effective. Our study, therefore, adds to the literature by pointing to the relevance of CBT associated with a rehabilitation program for children with epilepsy. Also, this study may underscore the role of EF in pediatric CBT and may point to the importance of EF in therapeutic interventions in children older than 9 years of age, even if they suffer from comorbid neurological deficits, including difficulties in setting goals and self-regulation impairments, which are quite prevalent.

A unique element of this study was its group intervention setting for children with epilepsy. The preliminary findings may suggest a need to widen protocols for treating psychosocial status among children with epilepsy by offering cost-effective means such as group therapy. Group therapies are thought to provide exposure to coping/problem-solving skills of peers who confront similar challenges, healthy attitudes, and peer support; thus, enabling mitigation of maladaptive strategies through mentoring and well-regulated peer feedback (70, 85, 86). Also, group sessions allow one to learn from the experiences of others, to better understand how to interact with people, and may serve as an initial friendly social network (46). Along with the benefits noted above, the group setting enabled a safe space in which to discuss one’s unique epilepsy and its impact on one’s personal life. In this regard future studies exploring group format efficacy in a neurological population and epilepsy specifically, are recommended.

### Limitations and Future Directions

In considering the implications of the current study, it is important to consider its limitations. Future work may include a larger sample size with a non-treated control group. Also, studying intervention efficacy on different neurological groups with memory deficits seems necessary to study protocol sensitivity and specificity.

Results point to the role of EF in improving verbal memory in this population. Future work may focus on language skills in this regard as well. Also, the intervention protocol included several compensating techniques that triggered prospective memory tasks. A clinical gap exists in tests for prospective memory, with no established means other than by self-report. Adding tools, such as Neuropage© or smartphones to the intervention may provide additional important data concerning specificity and generalization of the reported effects. Finally, interventions with children are recommended to address family issues, as the family often experiences stress associated with the child’s condition (87). Thus, adding estimations for parental support may be highly useful.

### Conclusion

This feasibility study indicates that a structured therapeutic rehabilitation program for a heterogeneous group of children with pharmacoresponsive epilepsy may improve both memory (visual and auditory verbal) and psychosocial aspects (sense of optimism and self-efficacy), using a cost-effective group setting. Additionally, results point to EF importance in memory rehabilitation, underscoring the importance of examining EF ability in intervention candidates and supporting EF abilities to ensure greater gains. Pending replication, current analyses underscore the benefits of considering and incorporating both cognitive and psychosocial modules; further, the analysis accounts for executive abilities in interventions models for children with epilepsy.

## Author Contributions

YS was responsible for literature search and the idea and execution of the entire design. RG guided, mentored, and inspired her in addition to taking a major role in writing and reviewing the manuscript. RC, AS, and BZ were consulted at each step of article preparation, writing, and corrections.

## Conflict of Interest Statement

There are no actual or potential conflicts of interest including any financial, personal, or other relationships with other persons or organizations that could inappropriately influence, or be perceived to influence this work.
